# A chromosomal analysis of *Nepa cinerea* Linnaeus, 1758 and *Ranatra linearis* (Linnaeus, 1758) (Heteroptera, Nepidae)

**DOI:** 10.3897/CompCytogen.v11i4.14928

**Published:** 2017-09-14

**Authors:** Robert B. Angus, Constance Jeangirard, Desislava Stoianova, Snejana Grozeva, Valentina G. Kuznetsova

**Affiliations:** 1 Department of Life Sciences (Insects), The Natural History Museum, Cromwell Road, London SW7 5BD, UK; 2 School of Biological Sciences, Royal Holloway University of London, Egham, Surrey TW20 0EX, UK; 3 Institute of Biodiversity and Ecosystem Research, Bulgarian Academy of Sciences, 1 Tsar Osvoboditel, Sofia 1000, Bulgaria; 4 Zoological Institute, Russian Academy of Sciences, Universitetskaya nab. 1, St. Petersburg 199034, Russia

**Keywords:** Karyotype, C-banding, (TTAGG)*_n_*, 18S rDNA, FISH, male meiosis, *Nepa cinerea*, *Ranatra linearis*, Nepomorpha, Heteroptera

## Abstract

An account is given of the karyotypes and male meiosis of the Water Scorpion *Nepa cinerea* Linnaeus, 1758 and the Water Stick Insect *Ranatra linearis* (Linnaeus, 1758) (Heteroptera, Nepomorpha, Nepidae). A number of different approaches and techniques were tried: the employment of both male and female gonads and mid-guts as the sources of chromosomes, squash and air-drying methods for chromosome preparations, C-banding and fluorescence *in situ* hybridization (FISH) for chromosome study. We found that *N. cinerea* had a karyotype comprising 14 pairs of autosomes and a multiple sex chromosome system, which is X_1_X_2_X_3_X_4_Y (♂) / X_1_X_1_X_2_X_2_X_3_X_3_X_4_X_4_ (♀), whereas *R. linearis* had a karyotype comprising 19 pairs of autosomes and a multiple sex chromosome system X_1_X_2_X_3_X_4_Y (♂) / X_1_X_1_X_2_X_2_X_3_X_3_X_4_X_4_ (♀). In both *N. cinerea* and *R. linearis*, the autosomes formed chiasmate bivalents in spermatogenesis, and the sex chromosome univalents divided during the first meiotic division and segregated during the second one suggesting thus a post-reductional type of behaviour. These results confirm and amplify those of Steopoe (1925, 1927, 1931, 1932) but are inconsistent with those of other researchers. C-banding appeared helpful in pairing up the autosomes for karyotype assembly; however in *R. linearis* the chromosomes were much more uniform in size and general appearance than in *N. cinerea*. FISH for 18S ribosomal DNA (major rDNA) revealed hybridization signals on two of the five sex chromosomes in *N. cinerea*. In *R. linearis*, rDNA location was less obvious than in *N. cinerea*; however it is suggested to be similar. We have detected the presence of the canonical “insect” (TTAGG)*_n_* telomeric repeat in chromosomes of these species. This is the first application of C-banding and FISH in the family Nepidae.

## Introduction

Heteropteran cytogenetics was reviewed by Ueshima (1979). He listed data on nine species of the water bug family Nepidae – three *Laccotrephes* Stål, 1866, one *Nepa* Linnaeus, 1758 (*N. cinerea* Linnaeus, 1758, listed as *N. rubra* Linnaeus, 1758) and five *Ranatra* Fabricius, 1790, including *R. linearis* (Linnaeus, 1758). The chromosome complements in males range from diploid numbers (2n) of 33 (*Nepa cinerea*) to 46 (*Ranatra chinensis* Mayer, 1865), and the sex chromosomes are listed as either XY or X_n_Y, or in one case X(0). Although the different sex chromosome systems are often recorded from different species, this is not always the case. Thus, *R. chinensis* is listed as having 2n = 46, comprising 44 autosomes plus XY sex chromosomes by Shikata (1949), but also as having 2n = 43 including 38 autosomes plus X_1_X_2_X_3_X_4_Y sex chromosomes by Ueshima (1979), using his own data. *Nepa cinerea* is listed by Spaul (1922) as having 2n = 35 (♂) with a simple sex chromosome system X(0) and 36 (♀), while the more extensive studies by Steopoe (1925, 1931, 1932) led to a male karyotype with 33 chromosomes, including 14 pairs of autosomes and X_1_X_2_X_3_X_4_Y sex chromosomes, a result supported by Halkka (1956). The only data listed for *Ranatra linearis* by Ueshima (1979) are from Steopoe (1927), who gives the chromosome complement as 2n = 43 (♂), including 19 pairs of autosomes and X_1_X_2_X_3_X_4_Y sex chromosomes. However, more recent work by Arefyev and Devyatkin (1988) based on the cell suspension preparation describes the complement as 2n = 46 (♂), postulating a simple sex chromosome system XY without any special arguments. Thus, there is either great variation between different populations of the above mentioned species, or some of the data might not be properly interpreted.

The early work on bugs was done using serial sections and this is also true of the objects of the present paper, the Water Scorpion *Nepa cinerea* and the Water Stick Insect *Ranatra linearis*. This technique can give very precise information on the orientation of the chromosomes in dividing nuclei and of the nuclei themselves within the tissues or organs (usually testes), but is of limited value in determining the sizes and shapes of the various chromosomes. Steopoe’s papers (1925, 1927, 1931) are particularly clear. For *N. cinerea* and *R. linearis* he shows both first and second male meiotic metaphases (MI and MII) with a ring of chromosomal elements round the edge of the metaphase plate, and a group of five chromosomes, arranged like the spots on a die, in the centre of the ring of chromosomes. The chromosome at the centre of this group is often the largest one. It has been demonstrated that, in this type of metaphase plate, the ring of chromosomes is made up of autosome bivalents (MI) or autosome univalents (MII), whereas the chromosomes in the centre behave as univalents (MI) or form a pseudobivalent / pseudomultivalent (MII) (Ueshima 1979). A striking feature of Steopoe’s work on both *Nepa* and *Ranatra* is that the median group of five appears much the same at both first and second meiotic metaphases. For this to be the case these chromosomes must be univalents and undergo an equational (mitotic) division during first meiosis. Steopoe interpreted these chromosomes as four X chromosomes assembled round a larger Y chromosome, and Halkka (1956) showed an early second anaphase in *N. cinerea* with one of the central elements moving to one pole and the other four to the other one. Neither Steopoe nor Halkka gave a female chromosome count, but for the system they describe to work, it has to be 2n = 36 in *Nepa cinerea* (as in Spaul 1922) and 2n = 46 in *Ranatra linearis*. Therefore, clear establishment of both male and female karyotypes should show which of the sex chromosome systems is present in these bugs.

The chromosomes in Heteroptera are holokinetic (Ueshima 1979). These chromosomes lack physical landmarks such as primary constrictions (the centromeres) and thus possess very few differentiating features. In recent years, different chromosome banding techniques (primarily C-, fluorochrome- and AgNOR-bandings) and Fluorescence *In Situ* Hybridization (FISH) have made it possible to get some chromosomal markers in karyotypes of Heteroptera (e.g., Grozeva et al. 2003, 2004, 2010, 2011, 2015, Angus et al. 2004, Waller and Angus 2005, Bressa et al. 2005, 2009, Angus 2006, Papeschi and Bressa 2006, Panzera et al. 2010, 2012, Poggio et al. 2011, 2012, 2013, 2014, Kuznetsova et al. 2012, 2015, Chirino et al. 2013, 2017, Chirino and Bressa 2014, Golub et al. 2015, 2016, Pita et al. 2016, Salanitro et al. 2017).

A prerequisite for good chromosome preparations is well spread cells with the chromosomes lying in one focal plane; however such cells are difficult to obtain using the squash method which is nowadays the most generally employed means of Heteroptera chromosome preparations. Besides, the use of this technique, which involves the placement of a cover slip over a tissue (usually testicular follicles) for flattening and spreading the chromosomes, can cause their damage and loss. Recently, a series of studies by Angus and co-authors (Angus et al. 2004, Waller and Angus 2005, Angus 2006) showed that an air-drying method combined with C-banding is a useful means of revealing cytogenetic markers allowing assembly of karyotypes from holokinetic chromosomes of several aquatic species, specifically of *Notonecta* Linnaeus, 1758 and *Corixa* Geoffroy, 1762 (Nepomorpha, Notonectidae and Corixidae, correspondingly).

In the present work we performed a detailed analysis of the karyotypes and male meiosis in *Nepa cinerea* and *Ranatra linearis* based on chromosome slides prepared from male and female gonads and mid-guts by air-drying and squash methods, including chromosome lengths and patterns of C-band distribution. Additionally, the work included the examination of the number and chromosomal location of major rDNA clusters and molecular structure of telomeres by FISH with 18S rDNA and the “insect” telomeric (TTAGG)*_n_* probes. This is the first employment of C-banding and FISH for the water bug family Nepidae.

## Material and methods

The localities (English and Bulgarian) from which the bugs were collected are given in Table 1.

The air-drying method of chromosome preparations and that of C-banding are as described by Angus et al. (2004). The living tissue was treated for 12.5 min in both the colchicine solution (0.1%) and the 0.5-isotonic KCl solution. C-banding was carried out on the 2-day old slides. Where slides had been Giemsa-stained and photographed under oil immersion, the oil was removed by immersion in xylene (2 changes, 5 min each) followed by 5 min in absolute ethanol. The slides were then destained by immersion in 2 × SSC for 10 min at 60°C and rinsed in unbuffered distilled water before the barium hydroxide treatment (about 8 min in saturated Ba(OH)_2_ solution at about 23°C, room temperature). The destaining in 2 × SSC may be unnecessary as R. Angus (unpublished data) now routinely C-bands Giemsa-stained slides of Coleoptera chromosomes, applying the Ba(OH)_2_ treatment to the slides once they have dried after immersion in absolute ethanol. The squash method of chromosome preparations and FISH procedure with 18S rDNA and (TTAGG)*_n_* probes were performed as described previously (Grozeva et al. 2011, 2015, Kuznetsova et al. 2012, 2015).

Giemsa stained and C-banded preparations were analysed under a Leitz Orthoplan microscope and photographed using a Wild MPS 51 camera and a Wild Photautomat MPS 45 with Kodak HQ high-contrast microfilm. Photographs were printed at 3000 × magnification, and then scanned into a computer where further manipulation and analysis of the images were done using Adobe Photoshop.

FISH preparations were analysed under a Leica DM 6000 B microscope, and images were acquired using a Leica DFC 345 FX camera and Leica Application Suite 3.7 software with an Image Overlay module.

The specimens from whom the chromosome preparations have been obtained are housed in R. Angus’ collection (Natural History Museum, London, UK) and at the Institute of Biodiversity and Ecosystem Research, BAS (Sofia, Bulgaria), correspondingly.

**Table 1.** Localities where *Nepa cinerea* and *Ranatra linearis* were collected.

**Species**	**Localities and number of specimens analysed**
*Nepa cinerea*	UK, Surrey: Ash, Lakeside Park (1♂, 1♀) 51.26°N 0.73°W
	Middlesex: Staines Moor (1 ♀) 51.52°N 0.52°W
	West Norfolk: Thompson Common (1♂) 52.52°N 0.82°E
	Bulgaria, Sofia: artificial lake in a park (1♂ juv.) 42.66°N 23.31°E
*Ranatra linearis*	UK, East Sussex: Pevensey Level (2 ♂♂) 50.81°N, 0.34°E
	Surrey: Runnymede, Langham Pond (2♀♀) 51.44°N, 0.56°W
	Bulgaria: Srebarna lake, (1♂ juv.) 44.10°N, 27.06°E.

## Results

### C-banding

***Nepa cinerea***, 2n (♂ / ♀) = 33 / 36 (14 AA + X_1_X_2_X_3_X_4_Y / X_1_X_1_X_2_X_2_X_3_X_3_X_4_X_4_)

**Figure 1.**
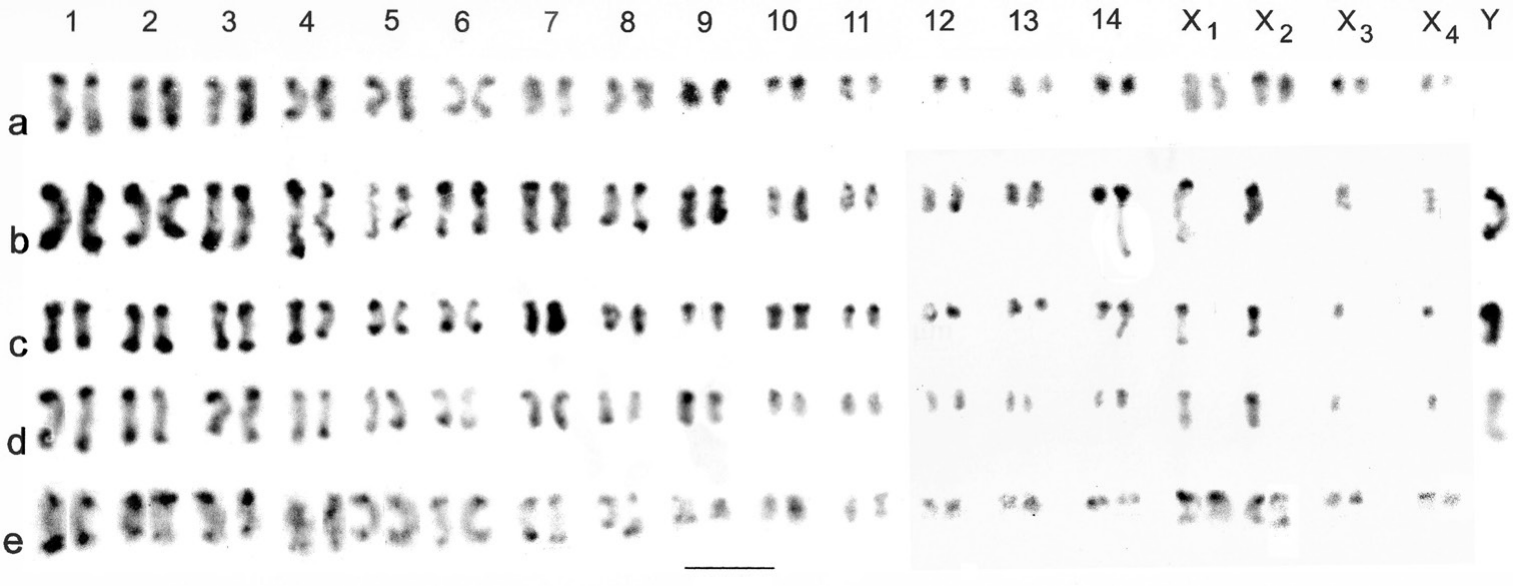
*Nepa cinerea*, C-banded mitotic chromosomes arranged as karyotypes. **a** ♀, ovary, Staines Moor **b** ♂, testis, Thompson Common **c, d** ♂, testis, Ash **e** ♀, mid-gut, Ash. Bar = 5 μm.

**Figure 2.**
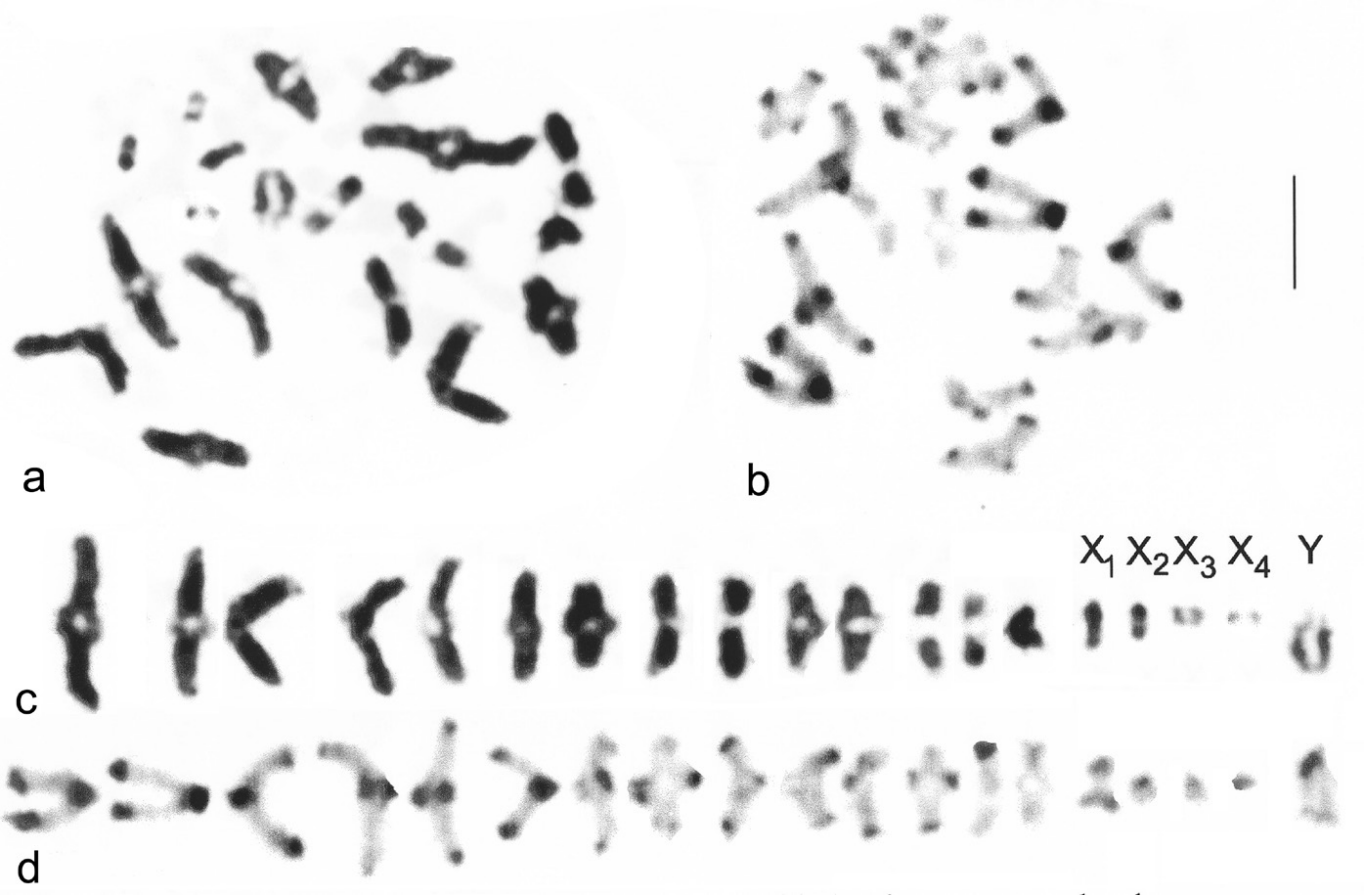
*Nepa cinerea*, ♂, Ash, first meiotic diakinesis/metaphase I from testis. **a, c** Giemsa-stained **b, d** C-banded. **a, b** nuclei as found **c, d** the same nuclei plated out and with the sex chromosomes labelled. Bar = 5 μm.

**Figure 3.**
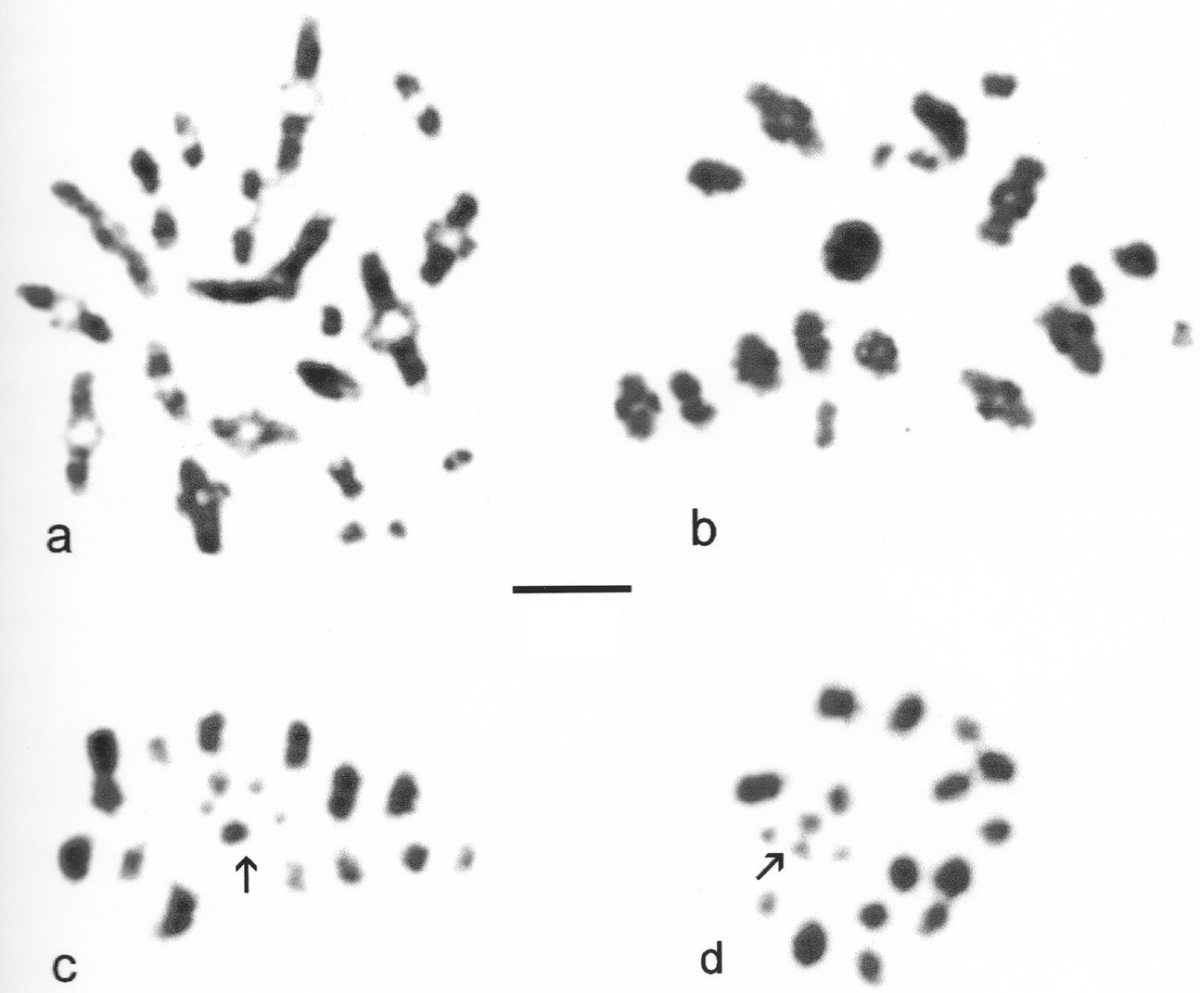
*Nepa cinerea*, ♂, Ash, first and second meiotic metaphases. **a, b** metaphase I **c, d** metaphase II. Sex chromosomes arrowed in **c, d**. Bar = 5 μm.

Male and female mitotic karyotypes (karyograms) are shown in Fig. 1a–e. First male meiosis is shown in Figs 2a–d and 3a, b, and second male meiosis is shown in Fig. 3c, d. Relative Chromosome Lengths (RCL, the length of each chromosome expressed as a percentage of the total haploid autosome length in the nucleus) are given in Table 2. Comparison of the C-banded karyotypes shows that the female (Fig. 1a, e) has four pairs of chromosomes which appear to be matched by single unpaired ones in the male (Fig. 1b-d), which also has a further large single chromosome. The large chromosomes, which are unpaired in the male but paired in the female, must be two of the four X chromosomes. The remaining unpaired chromosomes in the male are the large Y chromosome which has no counterpart in the female karyotype, and the two smaller ones which are taken as X_3_ and X_4_, but they are so small (the smallest chromosomes of the complement) that, on the present material, it is not possible to demonstrate that they are of the same or different sizes.

C-banding shows that the larger autosomes (pairs 1 – 4) have a distinct C-band at each end, but with some variation, possibly due to inadequacies of the C-banding method (Fig. 1). The C-banding pattern of the medium-sized autosomes (5 – 11) is variable, but the C-bands tend to be concentrated at one end and in the smaller autosomes, they are probably absent. Of the sex chromosomes, X_1_, X_2_ and Y have a strong C-band at one end while X_3_ and X_4_ have no clear banding. The banding of the autosomes in Fig. 1e reflects only partial success with the C-banding protocol.

The group of five chromosomes shown by Steopoe (1925, 1931, 1932) and Halkka (1956) as lying in the middle of the meiotic metaphase plate is very clear at second metaphase (Fig. 3c, d), but the position of these chromosomes is less distinctive at the first metaphase and diakinesis (Figs 2, 3a, b). It should be noted that the preparations figured here were made following colchicine treatment, which disrupts spindle formation, as well as cell-inflation by hypotonic saline. It is therefore not surprising that the orientation of the chromosomes is less clear than in the earlier work, which was done by serial sectioning. The arrangement of the sex chromosomes in metaphase plates of both divisions of meiosis shows that, while the autosomal bivalents separate and the homologs move to opposite poles of the spindle during first division, the sex chromosomes undergo an equational division at this stage. Thus, at second metaphase there is a ring of double-stranded autosomes which undergo an equational division and a group of single-stranded sex chromosomes, which segregate into four X chromosomes going to one pole and the Y chromosome which goes to the other one (the sex chromosomes post-reduction).

**Table 2.** Relative Lengths of *Nepa cinerea* chromosomes (measured in 3 males and 1 female).

Chromosome	RCL: mean (95% confidence intervals by t-test)	Number of measured cells
1	11.83 (10.86–12.80)	10
2	10.33 (9.79–10.87)	10
3	10.16 (9.68–10.64)	10
4	9.84 (9.28–10.40)	10
5	9.20 (8.16–10.24)	10
6	8.86 (8.13–9.59)	10
7	7.93 (7.61–8.25)	10
8	6.71 (6.10–7.32)	10
9	5.44 (4.76–6.12)	10
10	4.93 (4.32–5.54)	10
11	4.67 (4.29–5.05)	10
12	3.34 (2.81–3.37)	10
13	3.26 (2.75–3.77)	10
14	3.22 (2.56–3.88)	10
X_1_	7.39 (6.12–8.65)	7
X_2_	6.17 (5.57–6.77)	7
X_3_	3.14 (2.57–3.72)	7
X_4_	2.59 (1.92–3.25)	7
Y	9.80 (8.06–11.54)	3

***Ranatra linearis***, 2n (♂ / ♀) = 43 / 46 (19 AA + X_1_X_2_X_3_X_4_Y / X_1_X_1_X_2_X_2_X_3_X_3_X_4_X_4_)

**Figure 4.**
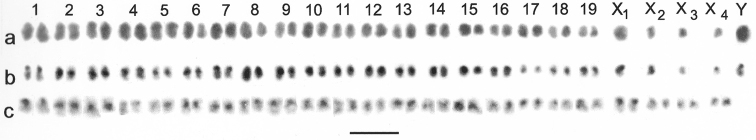
*Ranatra linearis*, mitotic chromosomes arranged as karyotypes. **a, b** ♂, Pevensey, testis **a** Giemsa-stained **b** the same nucleus, C-banded **c** ♀, Runnymede, mid-gut, C-banded. Bar = 5 μm.

**Figure 5.**
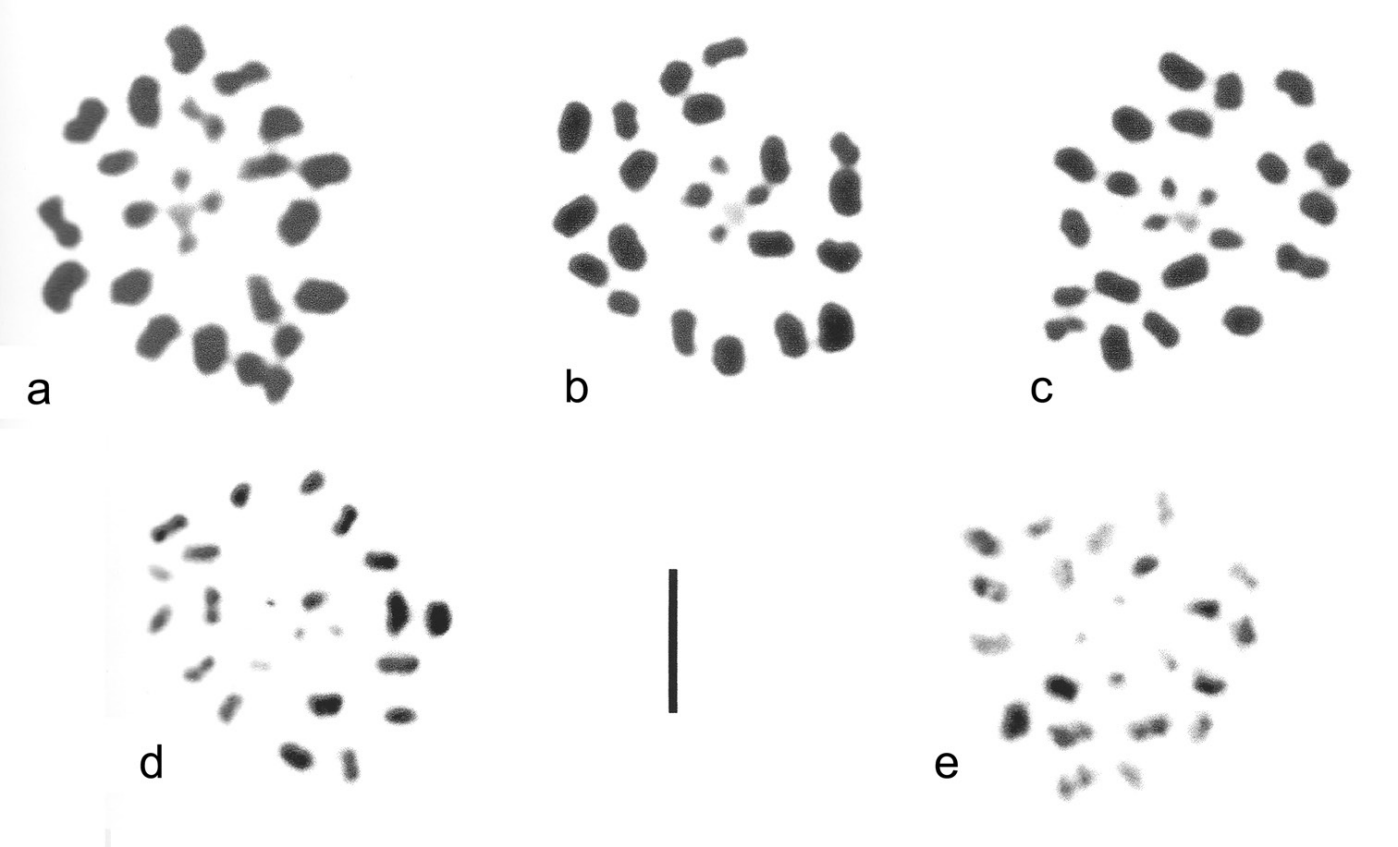
*Ranatra linearis*, ♂, Pevensey, meiosis **a–c** metaphase I **d, e** metaphase II. The central group of five chromosomes is very clear at first metaphase, but they have become displaced at metaphase II, especially in **e**. Bar = 5 μm.

Male and female mitotic karyotypes (karyograms) are shown in Fig. 4a–c. First metaphase of meiosis is shown in Fig. 5a–c and second metaphase in Fig. 5d, e. The karyotype includes 2n = 43 (♂) and 46 (♀). *R. linearis* has five more pairs of autosomes than *N. cinerea*, and the chromosomes are mostly smaller. The differences in chromosome length along the karyotype are less obvious than in *Nepa*, making the assembly of a karyotype more difficult. C-banding shows that nearly all the autosomes have one C-band at median, subterminal or terminal postions. Comparison of the karyotypes shown in Fig. 4a, b (unbanded and C-banded male mitotic chromosomes of the same nucleus) and Fig. 4c (C-banded female mitotic chromosomes) shows how C-banding can reveal more of the shape of individual chromosomes. Thus, the unbanded chromosomes tend to appear as elliptical masses but once C-banded they appear more rod-like. The pattern of sex chromosomes (X_1_X_2_X_3_X_4_Y), and their behaviour during the two divisions of meiosis, is the same as in *Nepa cinerea*. The arrangement pattern of the central group of five sex chromosomes is particularly clear in cells at metaphase I (Fig. 5a–c) and also in one cell at metaphase II (Fig. 5d), but they have been more disrupted by colchicine treatment and become displaced in other metaphases II (Fig. 5e). The general appearance of these sex chromosomes at the both metaphases is very similar, like the spots on a die. A similar resemblance of the general appearance of the sex chromosomes during first and second metaphases of meiosis has been shown by Suja et al. (2000) for species of the heteropteran families Pentatomidae, Pyrrhocoridae and Coreidae.

### FISH mapping of 18S rDNA and TTAGG telomeric repeats

**Figure 6.**
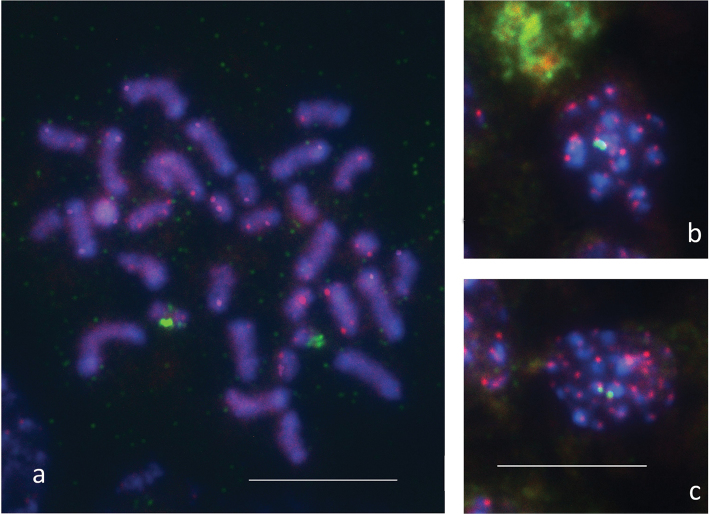
FISH with telomeric (TTAGG)*_n_* (red signals) and 18S rDNA (green signals) probes on mitotic chromosomes of *Nepa cinerea* (**a**) and meiotic chromosomes of *Ranatra linearis* (**b, c**). Two small signals (**c**) are united into one large signal (**b**). Bar = 10μm.

Figure 6a–c presents an example of the (TTAGG)*_n_* telomeric repeat distribution and major rDNA location at mitotic metaphase of a *N. cinerea* male (a) and at first metaphase (MI) of a *R. linearis* male (b, c), both males originating from Bulgaria. It is evident from the figure that the telomeric probe labels the ends of several chromosomes in both species indicating thus the presence of canonical pentameric insect telomeric repeats TTAGG in their genomes. It is interesting that in *N. cinerea* some of the larger chromosomes (with the heaviest C-bands) do not appear to show the telomeric signals. In *R. linearis*, with the meiotic metaphases, it does not seem possible to demonstrate with confidence the localization of the telomeric signals. In *N. cinerea*, FISH experiments with the 18S rDNA probe showed sharp and intense hybridization signals on two chromosomes, the signals being located at interstitial position on the larger chromosome and at terminal region on the smaller one. Since these chromosomes differ in size and rDNA clusters location, they are most likely either the X chromosomes (two of the four) or an X and the Y chromosomes. In *R. linearis*, the 18s rDNA probe identified two hybridization signals associated with two chromosomal elements of different size in the meiotic cells analysed (Fig. 6c). Based on the meiotic stages observed, we failed to determine the precise location of rDNA sites. Nevertheless, given that they are situated at one end of the chromosomal units, these are most likely univalents (i.e., sex chromosomes) rather than bivalents.

## Discussion

One of the first results to come from this work is to show how, in *Nepa cinerea* and *Ranatra linearis* from the Nepidae, as in species of other nepomorphan families, Corixidae and Notonectidae (Angus et al. 2004, Waller and Angus 2005, Angus 2006), the use of hypotonic-inflation and air-drying technique followed by C-banding, allows realistic karyotypes to be assembled. With holokinetic chromosomes, the only morphological characteristic available for karyotype production is chromosome length. Given that in most cases more than one pair of chromosomes will be more or less the same length, and that chromosomes show irregular condensation in individual preparations, it is not possible to produce convincing karyotypes using length alone. In Nepidae, the situation is made even worse because of the elliptical or blob-like appearance shown by many of the unbanded chromosomes. C-banding has the advantage of not only showing heterochromatic bands where they are present, but also of clearing the blob-like heavy staining of the chromosomes, so their actual shape becomes apparent. In *Nepa cinerea* the chromosomes are fairly long, the longer ones about 3μm in length, with C-bands at each end. These chromosomes tend to look distinctive, so that they can be paired up and karyotypes may be assembled with a fair degree of confidence, despite some ambiguity. The results from *N. cinerea* are useful as they show that the chromosome complement and sex chromosome arrangement described by Steopoe (1925, 1931, 1932) are in accordance with the cytogenetic description presented herein.

Spaul (1922) appears to have been mistaken about the sex chromosomes in this species, despite the apparent clarity of his drawings. However, Spaul is the only person to have published the female complement (2n = 36) – correctly. In his earlier papers, Steopoe (1925, 1931) used haematoxylin stained preparations and was concerned with the association between the chromosomes and the “plasmosome” (nucleolus) during meiosis. In particular, he thought that the association between the nucleolus and the group of five medially positioned sex chromosomes was the mechanism behind their positioning on the metaphase plate. Later, when he used Feulgen staining to show that the chromosomes and the nucleolus were chemically different (DNA and RNA) he attached less importance to this association (Steopoe 1932). The more recent observations of, e.g., Ueshima (1979), Nokkala (1986) and Kuznetsova et al. (2011, see also references therein) showed that in both meiotic metaphase plates involving the holokinetic chromosomes of heteropterans the autosomal bivalents/univalents (MI/MII) tend to form a ring round the edge of the plates whereas sex univalents and pseudobivalents/pseudomultivalents occupy the centre, which accounts very well for the arrangement shown by both *N. cinerea* and *R. linearis*.

The chromosomes of *R. linearis*, though amenable to the protocols used in this study, are both smaller and more numerous than those of *N. cinerea*, and the karyotype suggested has to be more tentative. However, the chromosome complement, with 19 pairs of autosomes, and sex chromosomes as in *N. cinerea*, is clear. One piece of new information in this study is the female karyotype of *R. linearis*, with three more chromosomes than the male, as in *N. cinerea*. The multiple sex chromosome system X_1_X_2_X_3_X_4_Y / X_1_X_1_X_2_X_2_X_3_X_3_X_4_X_4_ (male/female) found in these nepids stands in sharp contrast to the straightforward XY system found in Notonectidae and Corixidae (Ueshima 1979, Angus et al. 2004, Waller and Angus 2005, Bressa and Papeschi 2007). However, the multiple system may have originated from fragmentation of an original single (but large) X chromosome. Since the chromosomes are holokinetic, fragmentation does not result in loss of chromosome bits during cell division. The multiple sex chromosome systems, being found in species of Nepoidea and Ochteroidea, should be considered as derived characters within Nepomorpha (Bressa and Papeschi 2007).

One somewhat curious aspect of published work on the chromosomes of both *Nepa* and *Ranatra* is the two parallel views on the number of autosomes and sex chromosome mechanisms. Thus Spaul (1922) suggested diploid numbers of 35 (♂) and 36 (♀) for *N. cinerea*, with X(0) sex chromosome mechanism. For *R. chinensis*, Shikata (1949) reported the male complement with 46 chromosomes, 22 pairs of autosomes and XY sex chromosomes, but Ueshima (1979) claimed it had 43 chromosomes in the male, and sex chromosomes as described here for *R. linearis*, i.e., X_1_X_2_X_3_X_4_Y. The final twist to this tale comes from Arefyev and Devyatkin (1988), who report a complement of 46 chromosomes, including XY sex chromosomes, for male *R. linearis*. Sadly, they give no figure. It is at first sight impossible to reconcile these conflicting accounts. However, the detailed study of spermatogenesis in *N. cinerea* by Halkka (1956) may offer an explanation. Halkka observed that the division of the centrioles took place rapidly and early in the meiotic cycle and in some cases led to irregularities in chromosome division, with the production of polyploid and aneuploid spermatids. All previous work has been on testes, except, perhaps, for that of Arefyev and Devyatkin (1988) who did not know which tissues they were using as chromosome sources. However, in our study some karyotypes are from mid-gut cells, not subject to irregularities in spermatogenesis, so the results may be taken as correct.

A summary of all information on chromosome complements in *N. cinerea* and *R. linearis* derived from different studies conducted at different times by different investigators is presented in Table 3.

Another important result of this work is to show that the major rDNA loci are located on the sex chromosomes of *N. cinerea* and most probably also of *R. linearis* and that the ends of their chromosomes, the telomeres are composed of the pentanuceotide repeats TTAGG. These are the first data for the family Nepidae. In Heteroptera, there is a wide variation of major rDNA location: on different pairs of autosomes, on one or two sex chromosomes or on both autosomes and sex chromosomes, the differences being sometimes observed even between closely related, congeneric species (reviewed in Grozeva et al. 2015). Likewise, this is true of Nepomorpha, where in the two previously studied genera, *Belostoma* Latreille, 1807 and *Lethocerus* Mayr, 1853 (Belostomatidae), some species have 18S rRNA genes on autosomes while others on sex chromosomes (Papeschi and Bressa 2006, Kuznetsova et al. 2012, Chirino et al. 2013, Chirino and Bressa 2014). The data currently available are still so scarce and limited in their taxonomic representativeness that any speculation would be highly premature.

The TTAGG tandem sequence repeat found in our study in *N. cinerea* and *R. linearis* is considered the most typical and ancestral telomeric motif within the class (Sahara et al. 1999, Frydrychová et al. 2004, Vítková et al. 2005, Lukhtanov and Kuznetsova 2010, Chirino et al. 2017). Despite the widespread distribution of the (TTAGG)*_n_* motif among insects, it is not universally present in each order. For example, the huge order Coleoptera includes both TTAGG-positive and TTAGG-negative species, which has been interpreted as the multiple (at least eight times) loss of the initial telomeric sequence during beetle evolution (Frydrychová and Marec 2002, Mravinac et al. 2011). A similar heterogeneity is clearly exhibited also by Heteroptera with some species showing evidence for canonical telomeres and others not. The order comprises 7 infraorders and 40,000 species (Weirauch and Schuh 2011). The studies of telomeric DNA sequences were limited to 25 species, 17 genera and 9 families in the infraorders Nepomorpha (the families Belostomatidae and Nepidae; Kuznetsova et al. 2012, Chirino et al. 2017, present study), Gerromorpha (Gerridae; Mason et al. 2016), Cimicomorpha (Miridae, Cimicidae, Tingidae and Reduviidae; Frydrychová et al. 2004, Grozeva et al. 2011, Golub et al. 2015, Pita et al. 2016) and Pentatomomorpha (Pyrrhocoridae, Pentatomidae; Frydrychová et al. 2004, Grozeva et al. 2011). The (TTAGG)*_n_* telomeric sequence – according to our present knowledge – is present in both more basal infraorders Nepomorpha and Gerromorpha. Likewise, the (TTAGG)*_n_* motif is present in a sister to Heteroptera suborder Coleorrhyncha (Kuznetsova et al. 2015) and in several genera of Sternorrhyncha and Auchenorrhyncha (see for references Kuznetsova et al. 2015 and Pita et al. 2016). This indicates that it was most likely the ancestral telomere repeat sequence of Hemiptera as a whole. On the other hand, the ancestral motif (TTAGG)*_n_* was suggested to be lost in the early evolution of the evolutionarily derived heteropteran lineage composed by the sister infraorders Cimicomorpha and Pentatomomorpha being secondarily replaced by another motif or an alternative telomerase-independent mechanism of telomere maintenance (Frydrychová et al. 2004, Lukhtanov and Kuznetsova 2010, Mason et al. 2016). In all previously checked representatives of the families Miridae, Cimicidae, Tingidae, Pyrrhocoridae, and Pentatomidae the (TTAGG)*_n_* motif has not been found which supported well the above suggestion. Moreover, our dot-blot experiments have eliminated TTTTGGGG, TTGGGG, TTAGGC, TAACC, TTAGGG and TTTAGGG alternative variants as a potential replacement in tested TTAGG-negative species (Grozeva et al. 2011). Noteworthy in this context is a recent survey of sequenced genomes of several pentatomomorphan and cimicomorphan species confirming the lack of the TTAGG telomeric repeat and allowing suggestion that these groups have a defective version of telomerase gene (Mason et al. 2016).

However, a recent study of Pita et al. (2016) discovered unexpectedly the putative ancestral “insect” motif in the cimicomorphan family Reduviidae, namely in the youngest reduviid subfamily Triatominae, casting doubt on the above hypothesis since, according to the authors’ belief, “a new acquisition of the ancestral telomeric repeat in this recent evolutionary group is unlikely”. Moreover, the postulated lack of the (TTAGG)*_n_* detection in Cimicomorpha and Pentatomomorpha, by their hypothesis, “is due to a methodological problem of the telomeric probe rather than a loss process during their evolution”. We can not unconditionally agree with this view since in our studies, at least, the simultaneous labelling with the (TTAGG)*_n_* probe resulted in either a clearly defined or no FISH reaction in different species involved in the same experiment. To be sure, the absence of readable FISH signals in the particular taxa is not coincidental. One possibility is that in these taxa the TTAGG repeats are present but could not be localized by FISH due to their exclusively low amounts. It is our opinion that there still remains much work toward elucidating the problem and verifying the above hypotheses.

**Table 3.** A summary of data on karyotypes in *Nepa cinerea* and *Ranatra linearis*.

Taxon	Diploid	Haploid	References
Nepinae			
*Nepa cinerea*	35 ♂ 36 ♀ 33 ♂ 33 ♂ 33 ♂ 36 ♀	17AA + X(0) 14AA + X_1_X_2_X_3_X_4_Y* 14AA + X_1_X_2_X_3_X_4_Y* 14AA + X_1_X_2_X_3_X_4_Y 14AA + X_1_X_1_X_2_X_2_X_3_X_3_X_4_X_4_	Spaul 1922 Steopoe 1925, 1931, 1932 Halkka 1956 Present study
Ranatrinae			
*R. linearis*	43 ♂ 46 ♂ 43 ♂ 36 ♀	19AA + X_1_X_2_X_3_X_4_Y 22AA + XY 19AA + X_1_X_2_X_3_X_4_Y 19AA + X_1_X_1_X_2_X_2_X_3_X_3_X_4_X_4_	Steopoe 1927 Arefyev and Devyatkin 1988 Present study

*In Ueshima (1979) haploid complement of this species was erroneously presented as 19AA + X_1_X_2_X_3_X_4_Y
